# Epidemiological and molecular investigation of a rubella outbreak, Romania, 2011 to 2012

**DOI:** 10.2807/1560-7917.ES.2016.21.38.30345

**Published:** 2016-09-22

**Authors:** Mihaela Lazar, Emily Abernathy, Min-hsin Chen, Joseph Icenogle, Denisa Janta, Aurora Stanescu, Adriana Pistol, Sabine Santibanez, Annette Mankertz, Judith M Hübschen, Grigore Mihaescu, Gheorghe Necula, Emilia Lupulescu

**Affiliations:** 1 National Institute of Research-Development for Microbiology and Immunology “Cantacuzino”, Bucharest, Romania; 2 National Center for Immunizations and Respiratory Diseases, Centers for Disease Control and Prevention, Atlanta, Georgia, United States; 3 National Centre for Communicable Diseases Surveillance and Control, National Institute of Public Health, Bucharest, Romania; 4 WHO-EURO, Regional Reference Laboratory for Measles and Rubella, Robert Koch Institute, Berlin, Germany; 5 Department of Infection and Immunity, WHO-EURO Regional Reference Laboratory for Measles and Rubella, Luxembourg Institute of Health Esch-sur-Alzette, Grand-Duchy of Luxembourg; 6 Horia Hulubei National Institute for R&D in Physics and Nuclear Engineering, Bucharest, Romania; 7 University of Bucharest, Faculty of Biology, Department of Virology, Bucharest, Romania

**Keywords:** Rubella virus, Rubella genotypes, Rubella surveillance, Congenital rubella syndrome

## Abstract

We describe a rubella outbreak that occurred in Romania between September 2011 and December 2012. During this period 24,627 rubella cases, 41.1% (n=10,134) of which female, were notified based on clinical criteria, and a total of 6,182 individuals were found serologically positive for IgM-specific rubella antibody. The median age of notified cases was 18 years (range: <1–65) and the most affected age group 15 to 19 years (n=16,245 cases). Of all notified cases, 24,067 cases (97.7%) reported no history of vaccination. Phylogenetic analysis of 19 sequences (739 nucleotides each), from 10 districts of the country revealed that the outbreak was caused by two distinct rubella virus strains of genotype 2B, which co-circulated with both temporal and geographical overlap. In addition to the 6,182 IgM-positive rubella cases, 28 cases of congenital rubella syndrome (CRS) were identified, including 11 neonatal deaths and one stillbirth. The outbreak underscores the need to encourage higher vaccination uptake in the population, particularly in women of reproductive age, and to strengthen epidemiological and laboratory investigations of suspected rubella cases. Genetic characterisation of wild-type rubella virus is an essential component to enhance surveillance and here we report rubella virus sequences from Romania.

## Introduction

Rubella virus (RuV), the sole member of the Rubivirus genus in the Togaviridae family, is a positive strand RNA virus with a non-segmented genome of ca 9,762 nucleotides (nt). The genome encodes two non-structural (P90 and P150) and three structural (virion) proteins (the capsid and 2 envelope glycoproteins, E2 and E1). A 739-nt region between nt 8,731 and 9,469 within the E1 glycoprotein is the standard genotyping window for RuV [1,2]. Based on phylogenetic analysis of sequences of the structural protein coding region, two virus clades including a total of 13 genotypes, have been identified.

Infection with RuV generally leads to mild disease with symptoms that can include rash and low fever (<39°C) [3]. In pregnancy, however, RuV infection can cause miscarriages and serious birth defects including hearing, vision, mental, and heart impairment, which are collectively known as congenital rubella syndrome (CRS). CRS occurs in up to 85% of children born to women with RuV infection during the first trimester of pregnancy [4]. In addition, CRS can lead to neonatal deaths in up to 30% of cases [5].

Laboratory investigation plays an important role in both diagnosis and surveillance of rubella and CRS, since clinical diagnosis is unreliable and up to 50% of infections are estimated to be subclinical [6]. Typically, rubella is diagnosed by RuV specific IgM, but in pregnancy additional testing such as IgG avidity may be necessary.

False-negative rubella IgM can occur when specimens are taken within the first three days post-rash onset while false-positive IgM can result from cross reactions with rheumatoid factor or other viruses (such as parvovirus B19) [7,8]. In addition to serology, detection of viral RNA from nasopharyngeal swabs or oral fluid has been widely employed to confirm RuV infection. Moreover, polymerase chain reaction (PCR) can be used to obtain genetic information about circulating wild-type viruses to investigate transmission events [9,10].

When the European Region of the World Health Organization (WHO) adopted the goal of eliminating endemic rubella and measles by the end of 2015, the two key strategies were to achieve and sustain high vaccination coverage (≥95%) with two doses of measles, mumps, and rubella (MMR) vaccine and to strengthen surveillance systems through rigorous investigation and laboratory confirmation of outbreak-related and sporadic cases [11]. Because phylogenetic analysis of RuV genotypes can help determine whether circulating RuV strains result from endemic transmission or importations, laboratory surveillance for rubella also included the molecular characterisation of viruses. 

In Romania, selective vaccination for rubella and measles was offered to adolescent girls****aged****between 15 and 18 years (birth cohorts 1980 – 1983) as part of a mass vaccination campaign following a nation-wide measles outbreak in 1998 [12]. In 2004, MMR vaccination was introduced into the national immunisation programme with the first dose administered at 12 to 15 months of age and the second dose at seven years-old, and a rubella-containing vaccine was offered to girls aged between 13 and 14 years until 2008 (birth cohort 1994) [13]. Based on recent assessments of 18 month-old children however, the estimated MMR vaccine (one dose) coverage has decreased from 96.5% in 2010 to 89.3% in 2014 [14].

Rubella epidemics follow a 6 to 9 year cycle in the country. Between 2002 and 2003, Romania experienced a large rubella outbreak with more than 115,000 reported cases nationwide corresponding to an incidence of 549 cases per 100,000 population, the highest incidence ever observed in the 24 prior years [12]. In 2011 and 2012, another rubella outbreak occurred, with an incidence of 20.6 cases per 100,000 population in 2011 and 97.5 per 100,000 in 2012 [15]. This outbreak coincided with a measles outbreak, which took place between 2010 and 2013 and included 8,170 notified cases [16]. Here we provide an overview on the 2011 to 2012 rubella outbreak in Romania in terms of time, place and person, with a focus on laboratory and molecular analysis

## Methods

### Description of the surveillance systems

Since 1978 measles and rubella are statutorily notifiable diseases in Romania. Medical practitioners must report all possible measles or rubella cases to the regional public health authorities. The definition of a possible case in Romania concurs with the European Union (EU) case definition for possible cases and comprises any person with sudden onset of generalised maculopapular rash and at least one of the following five manifestations: cervical adenopathy, suboccipital adenopathy, post-auricular adenopathy, arthralgia, or arthritis [17,18]. 

A rubella surveillance system with case-based reporting with mandatory laboratory confirmation started in 2010. IgM antibody detection by enzyme-linked immunosorbent assay (ELISA) is the standard test for routine rubella surveillance recommended in the country [18]. In case of clusters/outbreaks, only five to ten sera from rubella possible cases are collected for testing [11,19]. 

Laboratory confirmation of cases in Romania is conducted according to a national methodology. Except for pregnant women, cases in Romania are either laboratory-confirmed by detecting rubella IgM antibodies in serum samples, or a significant rise in rubella IgG antibody levels, or PCR detection of RuV genetic material in nasopharyngeal swabs. In pregnancy, a rubella-specific IgG avidity test is additionally used to confirm rubella infection in rubella IgM-positive patients. Moreover, pregnant women, who are known to have been exposed to rubella, are assessed for rubella specific IgM and IgG antibodies and for those found to be negative another sample of serum is requested after 14 days to monitor IgM and/or IgG seroconversion [18].

As for measles, rubella surveillance is carried out among the general population, nationwide and all year round. The objectives of the surveillance are to facilitate the detection and laboratory confirmation of all possible sporadic cases, to identify chains of transmission and to investigate outbreaks. 

National surveillance for CRS, which is notifiable, was initiated in the year 2000 according to Romanian methodology. The clinical criteria for CRS apply to any infant < 1 year of age or any stillborn and include at least two of the following conditions: cataract(s), congenital glaucoma, congenital heart disease, loss of hearing, pigmentary retinopathy, or one of the above and either one of the subsequent manifestations: purpura, splenomegaly, microcephaly, developmental delay, meningo-encephalitis, radiolucent bone disease, or jaundice that begins within 24 hours after birth [20].

Infants who meet the CRS clinical criteria are usually investigated for rubella-specific IgM and IgG antibodies: a serum sample is collected as soon after birth as possible; for infants with IgM negative and IgG positive results, a second serum sample is required, according with the EU case definition [20].

### Collection and processing of samples 

From September 2011 to December 2012, within the routine surveillance system, the Romanian Public Health Districts collected 9,627 serum samples from possible rubella cases for laboratory confirmation. These 9,627 samples corresponded to 9,615 possible rubella cases, including 314 pregnant women (whereby two serum samples were respectively received from 12 pregnant women). 

During this time period 832 measles IgM-negative serum samples were also tested for rubella IgM. 

In accordance with the national surveillance for CRS, during the epidemic and post epidemic period (2012–2013) 178 serum samples were collected from 137 infants who met the clinical definition. 

From May 2011 to December 2012 (i.e. before and during the outbreak), 68 nasopharyngeal (NP) swabs from sporadic and outbreak-related cases were collected in different districts. Necropsy samples were obtained from one CRS case. 

Sera, swabs and necropsy samples were transferred for testing to the Cantacuzino Institute laboratory. Sera were maintained at 2–8 °C until testing (maximum of 6 days), then stored at –20 °C. The RNA extraction from swabs and the necropsy samples was done on the same day than the samples were received, followed by reverse transcription-(RT)-PCR detection, and in case of positive results by genotyping. The remaining swab samples and the extracted RNA were maintained at –70 °C.

### Serological assays

Detection of RuV specific IgM antibodies was performed using the Enzygnost Anti-Rubella Virus/IgM antibody enzyme immunoassay (EIA; Siemens, Marburg, Germany) or the Rubella virus IgM micro-capture EIA (IBL International). The Euroimmun Anti-Rubella Virus IgG and Avidity ELISA kit (Lubeck, Germany) was used for IgG and avidity testing. According to the manufacturer, relative avidity indexes are interpreted as follows: < 40% indicates low avidity antibodies and > 60% indicates high avidity antibodies, with 40–60% considered as intermediate (high avidity excludes rubella infection within the last 4 to 6 weeks before sample collection).

### RNA extraction from clinical specimens

In Romania, detection of RuV RNA or RNA extraction and subsequent genotyping were conducted only on NP swab samples and from necropsy samples (one case). 

As the number of swab samples collected during the outbreak was low (n=61), it was tested whether RNA could be obtained from IgM-positive serum samples that had been collected within three days after rash with a protocol used at the Centers for Disease Control and Prevention (CDC) in Atlanta, Georgia. Therefore in July 2014, 93 aliquots from such selected serum specimens were transported to the CDC for detection of RuV RNA and genotyping.

Total RNA was extracted from NP swabs with the Nucleospin Viral RNA kit (Macherey, Germany) according to the manufacturer’s instructions, except that 20 µL of proteinase K (20 mg/mL) was added in the lysis step and the RNA was eluted in 30 µL RNase-free H_2_O. 

RNA was also isolated from tissues (lung, kidney, spleen, lens, liver, brain, and thymus) from a deceased infant with CRS using TRIzol (Invitrogen, US). Extracted RNAs were stored at –70 °C.

For RNA extraction from sera shipped to CDC, the Qiagen ViralAmp RNA Mini kit (Qiagen, Valencia, CA) was used according to the manufacturer’s instructions. 

### Detection of rubella virus RNA

In Romania, two detection methods were used to detect rubella RNA in the clinical samples. Prior to 2012 a nested RT-PCR assay [21], which amplified a 143-nt region in the E1 coding region, was performed using GoScript Reverse Transcriptase and GoTaq Flexi DNA Polymerase (Promega,******Madison, WI, US) according to the manufacturer’s instructions, followed by gel electrophoresis. In 2012 a real-time RT-PCR assay for RuV RNA detection using the SuperScript III Platinum One-Step Quantitative RT-PCR System (Invitrogen, US) [22] was implemented. 

At CDC, a TaqMan real-time PCR assay targeting a 154 nt region near the 5’ end of the rubella genome and the same SuperScript kit was used (data not shown).

### Genotype determination

All genotyping assays were targeted to the RuV E1 coding region which contains the 739-nt region recommended by WHO for RuV genotyping. Generation of genotyping templates using RNAs from NP swab and tissue samples was performed by conventional RT-PCR reactions with the Qiagen OneStep RT-PCR Kit (Hilden, Germany) as described in Namuwulya et al. [11], except that the primers for the 5’ fragment were replaced by primers 8656F (5’-CCCCACCGACACCGTGATGAG-3’) and 9182R (5’-CGTGGATCCACTCGGGGATTT-3’). RNAs from sera which were positive by real-time RT-PCR were used as templates in one or more of three nested RT-PCR assays using specific primers pairs (Table). The nested assay 1 was used initially; samples that tested negative in this assay were subsequently tested using the assays 2 and 3.

**Table:** Primer sequences for three nested reverse transcription-polymerase chain reaction genotyping assays

Nested assay set number	Primer name	Primer sequence (5’–3’)	PCR product size	Nucleotides targeted
1	RV8633F	AGCGACGCGGCCTGCTGGGG	945	8,731–9,469
	RV9577R	CGCCCAGGTCTGCCGGGTCTC		
	RV8669F	GTGATGAGCGTGTTCGCCCTT	873	
	RV9541R	GTGTGTGCCATACACCACGCC		
2	RV8812F	CAACACGCCGCACGGACAAC	766	8,869–9,469
	RV9577R	CGCCCAGGTCTGCCGGGTCTC		
	RV8823F	ACGGACAACTCGAGGTCC	727	
	RV9541R	GTGTGTGCCATACACCACGCC		
3	RV8669F-2B	GTGATGAGCGTGTTCGCCCT	328	8,731–8,869
	RV8996R	CCACGAGCCGCGAACAGTCG		
	RV8691F-2B	CTAGCTACGTCCAGCACCC	271	
	RV8961R	CAAACCGGGGAGGCCCA		

PCR: polymerase chain reaction.

Sequences derived from assays 2 and 3 were combined to obtain the 739-nt sequence. All genotyping nested RT-PCR assays were performed with the Superscript III One-Step RT-PCR System with Platinum Taq High Fidelity DNA polymerase kit (Invitrogen) modified by the addition of betaine (Sigma, St. Louis, MO) to a final concentration of 1M. Cycling conditions for the first round consisted of one cycle of 30 min at 55 °C, 2 min at 94 °C, and 40 cycles of 10 s at 94 °C, 15 s at 55 °C, and 1 min at 68 °C. For the second round, 1 µL of the first round PCR was transferred and the 30 min at 55 °C RT step was eliminated. Negative and positive controls were carried through both rounds and master mix preparation and template addition were strictly separated. 

To sequence the DNA templates, the PRISM BigDye Terminator v3.1 Ready Reaction Cycle Sequencing kit (Applied Biosystems, Foster City, California) was used on a PRISM 3100-Avant Genetic Analyzer (Applied Biosystems).

### Phylogenetic analysis

The Romanian sequences were genotyped using the method recommended by the WHO [1]. GenBank accession numbers for the Romanian sequences are KP903737, KP903738, KP941058–62, KR021370–9 and KR054415–24. For phylogenetic analysis, an alignment was created and comprised 19 sequences from the 2011–2012 outbreak, three genotypes 1E and 1G sequences from the 2003–2004 outbreak, the 1E, 1G, and 2B WHO reference virus sequences and selected sequences from different parts of the world (26 2B sequences (2005–2014), seven 1G sequences (2003–2008) and two 1E sequences (2001–2003)). Searches to select the representative global strains were performed with basic local alignment search tool (BLAST) [23] and the selection was based on the degree of nt sequence homology with data from the Romanian outbreaks (≥ 99% identity), geographical distribution and collection date. Phylogenetic analysis was performed with the programme RAxML v8.00 [24] and the resulting tree was edited with the FigTree v1.4.2 programme [25] and the Inskape [26] programme for scalable vector graphics editing. The genetic distances were computed using the maximum-likelihood inference with generalised time-reversible (GTR) model of nt substitution and gamma model rate heterogeneity.

## Results

### Rubella incidence in Romania

**Figure 1:**
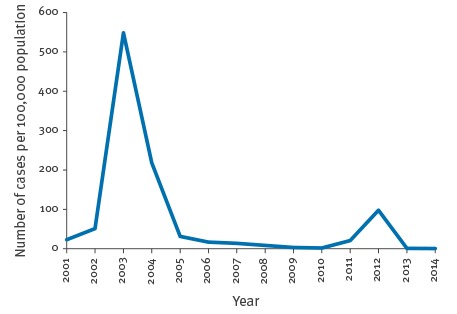
Rubella incidence in Romania, 2000–2014

After the year 2000, the incidence of rubella in Romania decreased following the 2003–2004 epidemic, from 218.5 cases per 100,000 population in 2004 to 1.6 in 2010 (Figure 1). In April and May of 2011, sporadic cases were notified in the south and south-east of the country. Subsequently in September, the outbreak started in the north-west, and spread further so that by the end of 2011 the total number of notified rubella cases amounted to 3,815 cases (18.2/100,000 population). In 2012, the whole country had become affected with 20,812 cases notified in that year (97.5/100,000).

### Description of the rubella outbreak

**Figure 2:**
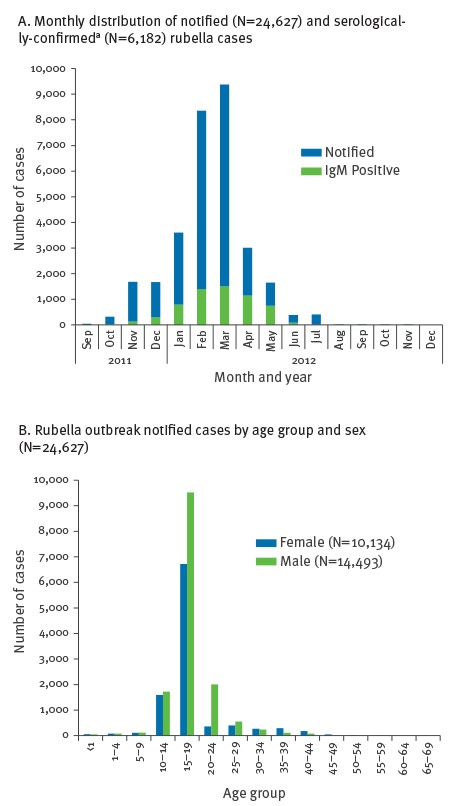
Distributions of notified rubella outbreak cases, Romania, September 2011–December 2012 (n = 24,627)

^a^ IgM positive.

From September 2011 to December 2012, a total of 24,627 cases were notified, 6,182 were confirmed (based on detection of IgM antibody), 18,442 were probable (based on an epidemiological link to a laboratory-confirmed case) and three were possible. Overall, 41.1% (n=10,134) of cases were of female sex and the median age was 18 years (range: <1–65), with the majority of cases (n=16,245) in the 15 to 19 year-old age group (Figure 2 A and B). Of all notified cases, 24,067 cases (97.7%) reported no history of vaccination, 528 cases (2.1%) reported one dose of MMR vaccine, and 23 cases (0.1%) reported two doses (vaccination histories were self-reported). For case-patients reporting vaccination, 114 (19.5%) were laboratory confirmed and 437 (80.5%) were considered probable by epidemiological link to laboratory-confirmed cases [15].

### Serological analysis

**Figure 3:**
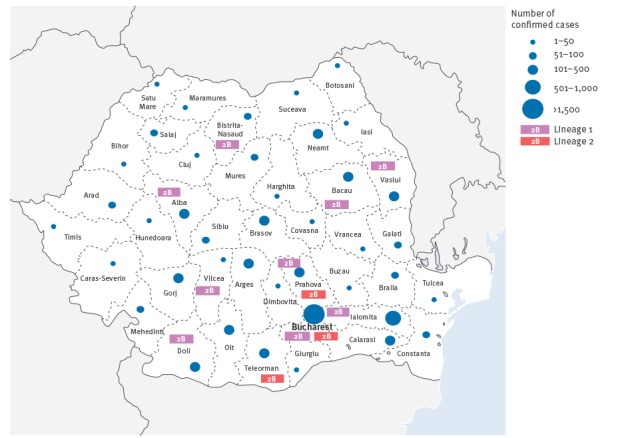
Geographical distribution of rubella serologically confirmed cases (n = 6,182)^a^ and virus genotype, Romania, September 2011–December 2012

^a^ Confirmed cases included 88 pregnant women and 5,820 other patients with rubella symptoms, as well as 274 patients suspected of measles but who, after being found negative for measles IgM, were found positive for rubella IgM.

Between September 2011 and December 2012, aside from sera obtained from 314 pregnant women (which are further described below), 9,301 serum samples were collected from possible cases of rubella and tested for the presence of rubella-specific IgM antibodies. Of these, 5,820 cases were positive for rubella IgM-specific antibody. Cases were from all parts of the country (Figure 3). Of the 3,481 IgM-negative serum samples, 1,726 (49.6%) were collected within 3-days post-rash onset. 

Serum samples received via the national measles surveillance programme, which were negative for measles specific IgM were also tested for rubella IgM. Between September 2011 and December 2012, 274 (30.3%) of the 832 measles IgM-negative serum samples, were positive for rubella-specific IgM.

### Rubella in pregnant women and congenital rubella syndrome cases

Sera from 314 pregnant women with clinical symptoms of rubella or known to have been exposed to rubella were tested for rubella-specific IgM and IgG antibodies. 

**Figure 4:**
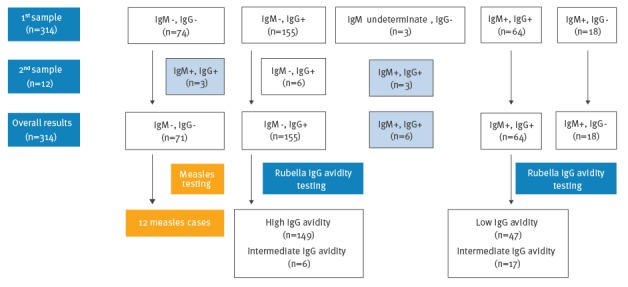
Serological testing of pregnant women with clinical symptoms of rubella or exposed to rubella, Romania, September 2011–December 2012 (n=314)

In a first respective serum sample, 232 pregnant women tested negative or indeterminate for IgM and 82 tested IgM positive. The 232 IgM-negative or indeterminate women consisted of 74 women negative for both IgM and IgG, 155 IgM-negative IgG-positive women, and three IgM-indeterminate IgG-negative women. The 82 IgM-positive pregnant women comprised 18 women testing IgM positive IgG negative and 64 testing IgM positive IgG positive (Figure 4). 

IgG-: IgG negative; IgG+; IgG positive; IgM-: IgM negative; IgM+: IgG positive.

The boxes shaded in light blue, in the respective 2^nd^ sample and in overall result rows, highlight samples with evidence of seroconversion.

Follow-up samples for further laboratory confirmation could not be obtained for all pregnant women, however 12 women with negative or indeterminate IgM results were retested on a second sample received 14 days after the first. Six of these 12 women were initially among the 155 IgM negative IgG positive women, three were initially IgM indeterminate IgG negative, and three were initially part of the 74 women negative for both IgM and IgG. The first six women’s tests remained unchanged in the second sample (i.e. still IgM negative, IgG positive, and with an intermediate IgG avidity), while for the latter six there was evidence of seroconversion, as they tested positive for both IgM and IgG in the second sample.

In total, 88 pregnant women were found to have rubella specific IgM-antibodies. The remaining rubella IgM-negative sera were subsequently tested for measles-specific IgM antibodies and 12 pregnant women were determined to be measles cases.

Overall, the number of women who tested positive for rubella-specific IgG only (i.e. IgM negative, IgG positive) amounted to 155. All had IgGs tested for avidity, and 149 were found with high avidity IgG antibodies, while six had intermediate avidity IgGs. 

Of the total 88 IgM positive pregnant women, six could be confirmed as rubella cases by evidence of seroconversion in the second serum sample. For the 64 IgM-positive women who were IgG positive in the first sample, IgG avidity testing was conducted, whereby 47 had low and 17 intermediate avidity IgG, confirming primary rubella infection. Because a second serum sample could not be obtained from 18 women with initial IgM positive IgG negative results, IgG avidity testing was not possible for these persons. Taking into account their symptoms and the epidemiological context however, they were nevertheless included as outbreak cases. 

When available, IgG avidity was used as a complementary test to the IgM antibody results, to determine the possible timing of contracting rubella. Based on this approach, it was estimated that 25 pregnant women (28.4 %; 25/88) were likely infected during the first trimester. A total of 14 pregnancies were terminated.

Serum samples from 137 infants suspected of having been exposed to RuV during fetal development were collected, 38 were IgM positive. In addition, RNA was also collected from a stillborn infant. Combined with clinical criteria, 27 infants were laboratory-confirmed to be CRS by IgM and IgG testing, while the stillborn was confirmed to have been infected by RuV using PCR. The other 11 infants were identified to have congenital rubella infection by an IgM-positive test at birth, and an epidemiological link (the mother was confirmed with rubella infection during pregnancy) but without observable defects. Such children are not followed-up. Of 28 infants with CRS, one was a stillbirth and 11 died after birth.

Formalin-fixed, paraffin-embedded (FFPE) tissue specimens obtained from one confirmed child with fatal congenital rubella autopsy were submitted to the CDC for additional studies (histopathological and immunohistopathological evaluation) [27]. 

### Reverse transcription-polymerase chain reaction and genotyping

In May of 2011, two sporadic rubella cases in Bucharest were confirmed. Both cases occurred three months before the outbreak was recognised and had no recent history of travel. A virus sequence from one of these sporadic cases (RVs/Bucharest.ROU/18.11) was determined to be genotype 2B. Between May 2011 and December 2012, 68 NP swabs were collected from cases occurring in 21 of 42 districts. Thirty-three (48.5%) swabs were positive for RuV RNA by either the nested or real-time RT-PCR assay. Of these, PCR templates for genotyping were generated from 11 swab samples (36.4%). 

In addition, RNAs from necropsy tissues (lung, kidney, spleen, liver, brain, thymus and lens) from one case were positive for rubella by real-time RT-PCR and RNA from the kidney was used to genotype the virus. 

RNA was also extracted from 93 IgM-positive sera which were collected three days after rash onset or earlier. Of these, rubella RNA was detected by real-time RT-PCR in 20 sera (21.5%); the average cycle threshold value was 37 of 40 cycles (range: 35.7–39). Genotypes were determined from seven sera (7.5%). Three of the RNAs derived from serum were amplified by nested primers set 1 and four required the amplification of both the nested primer sets 2 and 3 to obtain the 739-nt sequence. 

In total, 19 sequences were obtained from rubella cases between May and December 2012, representing samples from 10 distinct districts (Figure 3). The genotype of all the sequences was determined to be 2B by comparison to the WHO reference sequences (data not shown).

**Figure 5:**
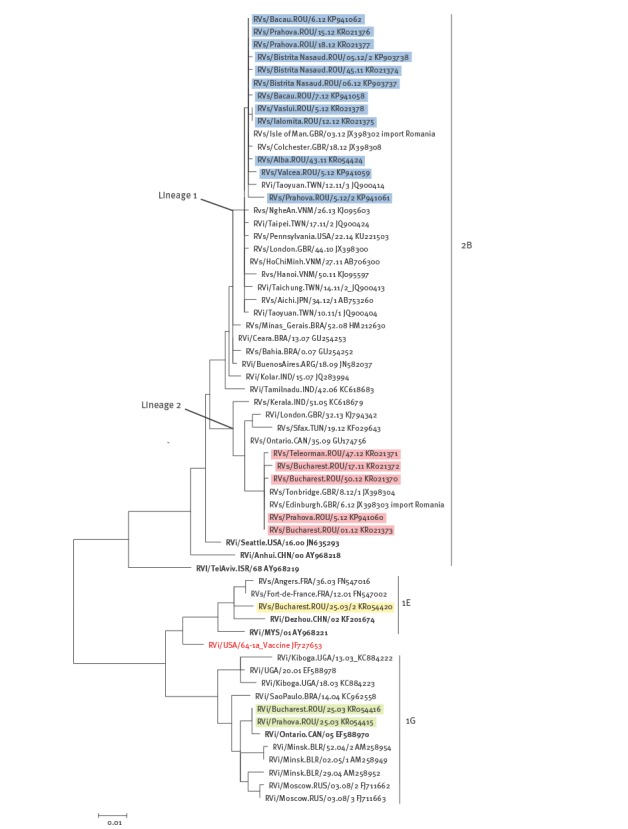
Phylogenetic analysis of sequences from rubella viral strains retrieved in Romania in 2003 and 2011–2012

In order to compare the sequences from the 2011–2012 outbreak to earlier sequences from Romania and sequences of the same genotypes retrieved worldwide, a phylogenetic tree of genotypes 1E, 1G, and 2B is shown (Figure 5). The 2003 1E and 1G sequences (in yellow and green in Figure 5; Robert Koch Institute, Berlin) from two Romanian cities, Bucharest and Prahova, were found in the same clusters as viruses from other European countries from the same time period (e.g. for 1E, RVs/Angers.FRA/36.03; for 1G, RVi/Minsk.BLR/52.04/2). The 2B sequences from the 2011–2012 Romanian outbreak assort into two lineages with 3.11–3.92% nt (23–29 nt) difference between the two lineages. Lineage 2 appeared to have had a smaller geographical range, being found in Bucharest and two other districts, while lineage 1 was found in Bucharest and seven additional districts (Figure 3). Lineage 1 contains 12 of the Romanian sequences from 2011 and 2012 as well as 2010–2014 sequences from different areas of Asia including Japan, Taiwan and Vietnam. One of three sequences from Great Britain in this cluster (RVs/Isle of Man.GBR/03.12) was epidemiologically linked to an importation from Romania (Kevin Brown, personal communication, 25 January 2016). This lineage descends from sequences from South America and India detected from 2006 to 2009. Lineage 2 contains seven of the Romania sequences, including a sequence from a sporadic case early in 2011 (RVs/Bucharest.ROU/18.11) and six from 2012. Lineage 2 also contains another import into Great Britain from Romania (RVs/Edinburgh.GBR/06.12) (Kevin Brown, personal communication, 25 January 2016). Other sequences from the same time period as the Romania outbreak in this lineage are from Tunisia, and Great Britain, with two older sequences from Canada (2009) and India (2005).

Romanian sequences from 2003 are highlighted in yellow (1E) and green (1G) while those from 2011–12, are highlighted in blue (2B lineage 1), and red (2B lineage 2). The sequences are according to the World Health Organization recommended standard 739-nt window in the envelope (E)1 coding region of the rubella virus genome, and are compared with WHO reference strains (in bold) and representative sequences from RuV genotypes 1E, 1G, and 2B.

## Discussion

Rubella is usually a mild benign disease, but due to its devastating effects in pregnancy, control and elimination programmes have been instituted in many countries; the disease has been eliminated by immunisation programmes in several countries, including those in the WHO Region of the Americas [28-31]. Universal rubella vaccination of one year-old infants was implemented in Romania in 2004; however, outbreaks continue to occur following a typical 6 to 9 year epidemic cycle. The total number of cases notified in Europe since 2007 varied from 26,827 in 2007 to 4,767 in 2010 then increased to 8,318 in 2011 and to 26,014 in 2012 [30,32]. In 2011, 97% of the rubella cases in Europe were reported from Poland and Romania [33], although it has to be taken into account that rubella surveillance has not been implemented in all European countries. During the 2011–2012 outbreak in Romania, cases occurred in all the districts of the country, amounting to 24,627 notified cases, most of which were unvaccinated (97.7%). The majority of cases were 15 to 19 year-olds who were missed by the current vaccination strategy (the MMR coverage among adolescents is not routinely monitored in Romania). The 2011–2012 outbreak resulted in the birth of 28 children with CRS, including 11 deaths and one stillbirth.

In Romania, rubella surveillance requires laboratory detection of IgM-specific antibody in serum collected from each sporadic case and the first cases from rubella outbreaks [11]. A limitation of rubella IgM tests, however, is that the IgM response may not have developed for a serum collected within the first 72 hours after rash onset, resulting in a false-negative result [22,34]. In the 2011–2012 outbreak 3,481 serum samples were negative for specific rubella IgM, but 49.6% of the negative sera were collected too close in time to the onset of rash; therefore, the total number of serologically-confirmed cases (n = 6,182) in the outbreak was likely underestimated.

In addition to serological testing, molecular detection of RuV RNA is useful for the further confirmation of rubella infection, especially in the five days after rash onset [22]. Moreover, sequence information can be obtained and used to differentiate between vaccine and wild-type infections and, in combination with well-established baseline genetic and epidemiological data, to identify indigenous or imported viruses. The ideal samples for rubella isolation and detection are NP specimens, collected as soon as possible after the onset of symptoms (< 5 days after rash onset), but collection of samples for virological surveillance can be challenging due to the logistics of storage and transport. Nevertheless, we were able to obtain 68 swabs and genotype 11 samples. In addition, although sera are not optimal specimens for viral detection due to the low amounts of viral RNA present, RuV RNA was detected in 21.5% of IgM positive serum samples collected close to symptom onset, while genotypes were obtained from 7.5%. These numbers are in good agreement with a previous study of RuV RNA in sera in which 26% of sera samples were real-time PCR positive and 12% yielded genotypes [35].

Genetic information obtained from the 2011–2012 outbreak in Romania revealed that it was driven by two 2B lineages with an average of 3.5% nt difference, which overlapped both temporally and geographically. Data from other countries have shown that co-circulation of multiple RuV lineages of one genotype within a country is quite common [36,37]. Lineage 1 Romanian outbreak sequences, which were most similar to those of viruses from south-east Asia, were detected from late 2011 through the spring of 2012 while lineage 2 sequences, which were most similar to a viral strain from northern Africa, were detected in May 2011 from one of the sporadic cases and then from early 2012 through December of 2012. In addition, analysis of sequence data confirmed that viruses which were identified by epidemiological data as exportations to Great Britain were identical to viruses from Romania.

The viruses of genotypes 1E and 1G detected in Romania in the previous outbreak in 2003 were not detected in the 2011–2012 outbreak and no sequences from Romania are available from the intervening time period; therefore, it is not possible to know when the genotype 2B viruses entered the country. The viruses in the 2011–2012 outbreak may have been circulating in Romania before the outbreak. However, they may have been introduced by recent importation events, as would be suggested by the high degree of sequence similarity with viruses from approximately the same time period (2011 to 2013) detected in other parts of the world such as Japan, Vietnam and Taiwan (Figure 5). The very low incidence of rubella in the two to three years before the outbreak is consistent with this hypothesis. In addition, the approximate nine month gap that elapsed between the first and second detections of lineage 2 suggests that there may have been two separate introductions of this lineage. Gaps in viral surveillance for rubella both regionally and globally limit the ability to use genetic data for identifying the source of a particular lineage. It is clear, however, that a shift occurred in the RuV genotypes over time from genotypes that were common in other European countries (1E and 1G) to the 2B genotype that was previously found primarily in other parts of the eastern hemisphere [1]. Such genotype shifts have been documented in other countries such as China [35] and Brazil [38].

This is the first report of RuV sequences from Romania. Documenting virus genotypes is one of the essential criteria for tracking the progress of elimination of rubella in the WHO European region [39]. Thus, determining the endemic RuV genotype baseline is necessary. However, as shown here circulating genotypes can change over time and ongoing surveillance is necessary to provide up-to-date information. 

In order to reach the goal of endemic rubella elimination and, thus, prevent CRS cases such as those that resulted from the 2011–2012 outbreak, it is necessary to achieve and sustain high vaccination coverage > 95% (the women in reproductive age who were born before 2004 should be better informed on the risks of rubella, encouraged to get vaccinated and women in childbearing age checked for immunity to rubella prior to pregnancy) supported by high-quality surveillance including epidemiological, serological and molecular studies. This process will include the development of case-based epidemiology investigations to identify importations and prevent secondary transmissions especially in countries such as Romania where rubella virological surveillance is not yet well established.
